# Familial SLC29A3-related histiocytosis with presumed choroidal infiltration: expanding the spectrum of histiocytosis-lymphadenopathy plus syndrome

**DOI:** 10.1016/j.ajoc.2026.102570

**Published:** 2026-03-20

**Authors:** Xavier Boulu, Gilles Morin, Christophe Attencourt, Jean Schmidt, Thi Ha Chau Tran

**Affiliations:** a Department of Internal Medicine, Amiens University Medical Center, F-80054, Amiens, France; b Department of Medical Genetics, Amiens University Medical Center, F-80054, Amiens, France; c Department of Pathology, Amiens University Medical Center, F-80054, Amiens, France; d Department of Ophthalmology, Amiens University Medical Center, F-80054, Amiens, France

**Keywords:** H syndrome, Uveitis, Choroidal infiltration, Non-Langerhans histiocytosis, SLC29A3, ENT3

## Abstract

H syndrome (HS) is a rare autosomal recessive histiocytosis caused by biallelic mutations of the *SLC29A3* gene. Ophthalmological involvement is not typical in HS, but is a known manifestation of non-Langerhans histiocytoses. We report two adult siblings with genetically confirmed HS who developed bilateral choroidal infiltration, expanding the phenotypic spectrum of SLC29A3-related histiocytosis.

The first case was a woman with a history of HS who presented with progressive visual loss and choroidal lesions on imaging. Systemic findings and histological analyses confirmed non-Langerhans histiocytosis without BRAF V600E mutation. This patient's brother, who was previously asymptomatic, had similar choroidal and systemic findings. Both displayed evidence of MAPK pathway activation (phospho-ERK positive) without detectable somatic mutations.

These findings expand the phenotypic spectrum of SLC29A3-related histiocytosis to include presumed choroidal involvement.

## Highlights

• Familial SLC29A3 histiocytosis with bilateral choroidal infiltration.
• Multimodal imaging reveals a thickened, disorganized choroid.
• Phospho-ERK–positive lesions without somatic MAPK variants.
• MEK inhibition stabilizes visual and systemic disease.

## 1 Introduction

H syndrome (HS) is an autosomal recessive histiocytic disorder caused by biallelic mutations of the *SLC29A3* gene,^1^ which encodes equilibrated nucleoside transporter 3 (ENT3).

ENT3 is localized in intracellular organelles — primarily lysosomes, endosomes, and mitochondria — and facilitates nucleoside transport across the membranes of these organelles. The loss of ENT3 function disrupts lysosomal homeostasis, leading to nucleoside accumulation and abnormal immune activation. In *SLC29A3*^*−/−*^ mouse models,^2^ ENT3 deficiency results in the systemic infiltration of CD68^+^ CD163^+^ histiocytes and is associated with an upregulation of the mechanistic target of rapamycin (mTOR) signaling pathway. This promotes macrophage proliferation and tissue infiltration, contributing to the multisystemic manifestations of the syndrome.

This genodermatosis, first described in 2008,^3^ is predominantly observed in consanguineous Arab populations.^4^ Fewer than 100 cases have been reported.^4^ Clinically, H syndrome combines dermatologic and systemic features with an onset during the first two decades of life.^4^^,^^5^ These features include linear hyperpigmented scleroderma-like lesions on the lower body, with hypertrichosis, heart abnormalities, hepatosplenomegaly, hearing loss, hypogonadism, short stature, hyperglycemia (diabetes mellitus), and camptodactyly.

Histological findings in H syndrome^4^ typically include a dense dermal infiltrate of CD68^+^ CD163^+^ histiocytes, negative for CD1a and Langerin, associated with variable fibrosis and scattered lymphocytes. The absence of BRAF V600E mutation and the non-clonal nature of the infiltrate distinguish this syndrome from Erdheim-Chester disease, which has overlapping features.^6^^,^^7^

Ophthalmological involvement is uncommon in HS^8^ but is a known manifestation in several non-Langerhans histiocytoses^9^, ^10^, ^11^, ^12^, ^13^. We report the case of two siblings with genetically confirmed biallelic pathogenic SLC29A3 variants who developed presumed choroidal infiltration in adulthood, expanding the phenotypic spectrum of SLC29A3-related histiocytosis.

## 2 Case 1

**Fig. 1:**
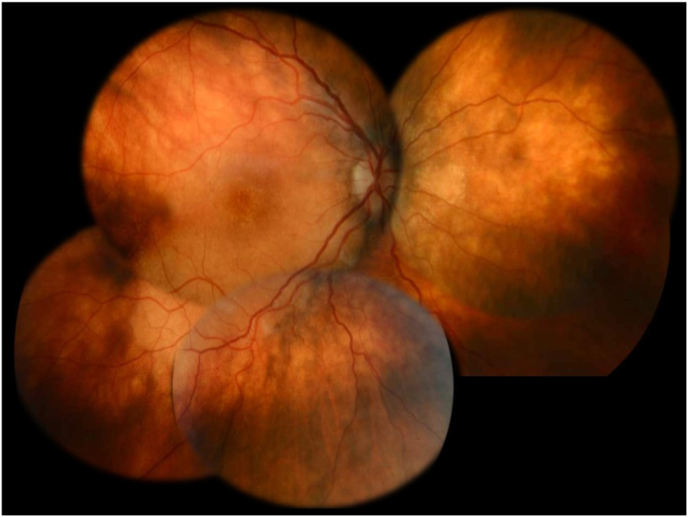
Fundus photograph of the right eye showing a yellowish subretinal lesion in the posterior pole and juxtapapillary area of the nasal quadrant.

**Fig. 2:**
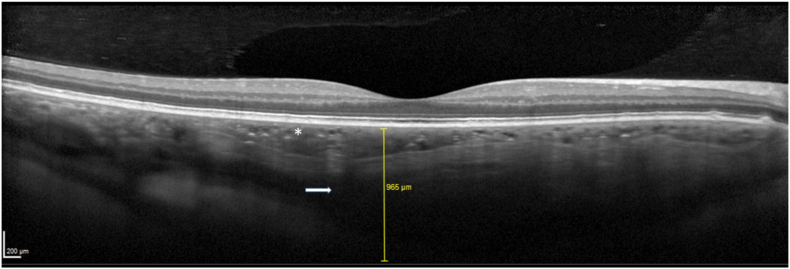
Thick disorganized choroid with compressed choriocapillaris and a disorganization of choroidal architecture. Subfoveal thickness >900μm.

A 25-year-old woman was referred for ophthalmological evaluation. Her best-corrected visual acuity was 20/20 for both eyes. The spherical equivalent refractive error (SER) was −1.5 D for the right eye (OD) and −0.75 D for the left eye (OS). Fundus examination revealed a yellowish, creamy choroidal infiltration without retinal pigment epithelium (RPE) elevation in the posterior pole and juxtapapillary area of the nasal quadrant (Fig. 1). The far peripheral retina was unremarkable. Optical coherence tomography (OCT) demonstrated a normal foveolar profile with clear retinal layers. Subfoveal choroidal thickness exceeded 900 μm on OCT. A hyporeflective band was observed beneath the RPE, extending to the choroid–scleral junction within Haller's layer, suggesting disruption of the choroidal architecture. The choriocapillaris appeared thin and compressed (Fig. 2).

**Fig. 3:**
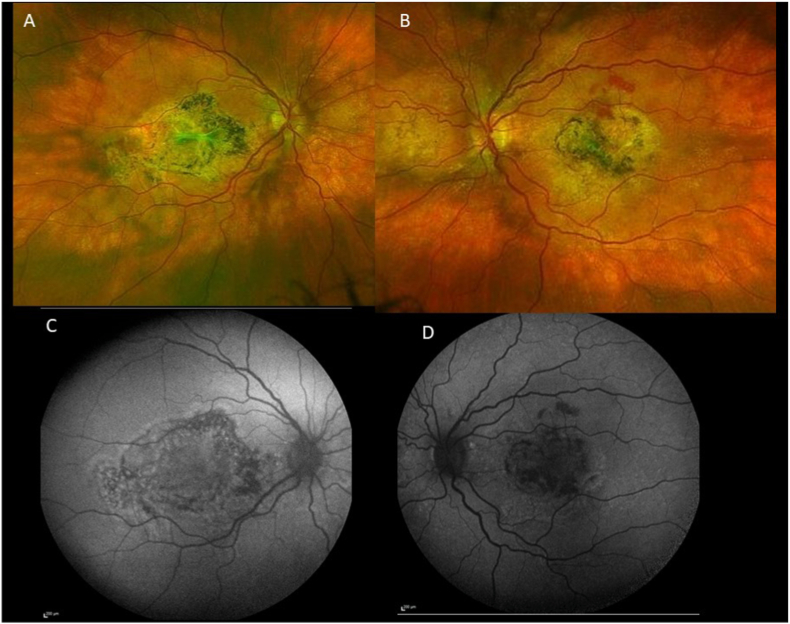
Fundus color photographs of the right eye (A) and the left eye (B) showing areas of retinal atrophy and RPE elevation. Fundus autofluorescence of the right eye (C) and the left eye (D) revealing hypofluorescence due to the masking effect of RPE pigmentation and the surrounding hyperautofluorescence. (For interpretation of the references to color in this figure legend, the reader is referred to the Web version of this article.)

**Fig. 4:**
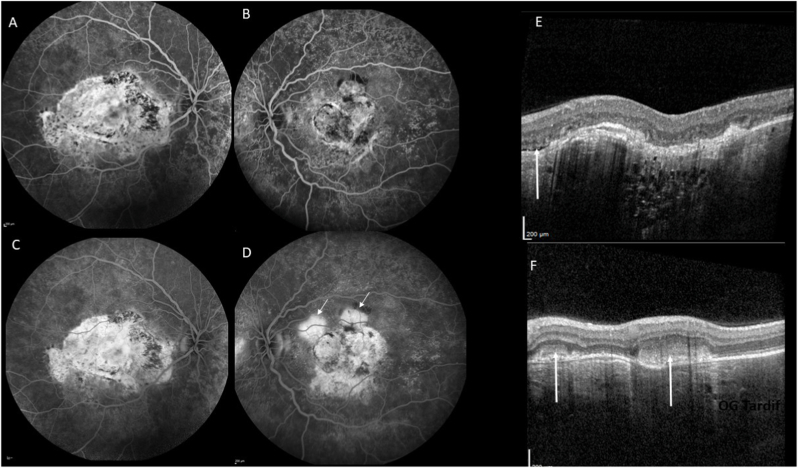
Fluorescein angiography of the right eye (A: early phase, B: late phase) and the left eye (C: early phase, D: late phase) showing heterogeneous hyperfluorescence with staining in the right eye and leakage (arrows) in the left eye. OCT scans showing the presence of subretinal fluid (E, arrow), a dome-shaped elevation of the choroid in the posterior pole, and a hyperreflective subretinal exudation (F, arrows) at the site of choroidal neovascularization.

**Fig. 5:**
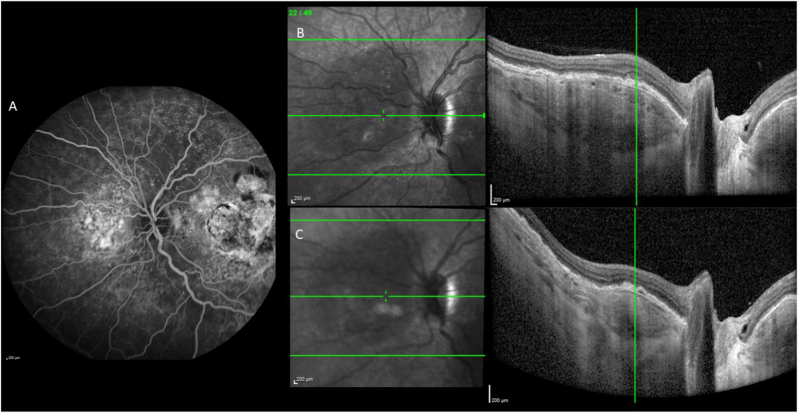
Fluorescein angiogram of the juxtapapillary area of the nasal quadrant (A) showing ill-defined hyperfluorescence. OCT scan (B) showing a dome-shaped elevation of the choroid, effacement of the choroidal vessels , a disorganization of choroidal architecture, and an enlarged Haller vein bordering the infiltrate. Choroidal thickness decreased under MEK inhibition (C).

**Fig. 6:**
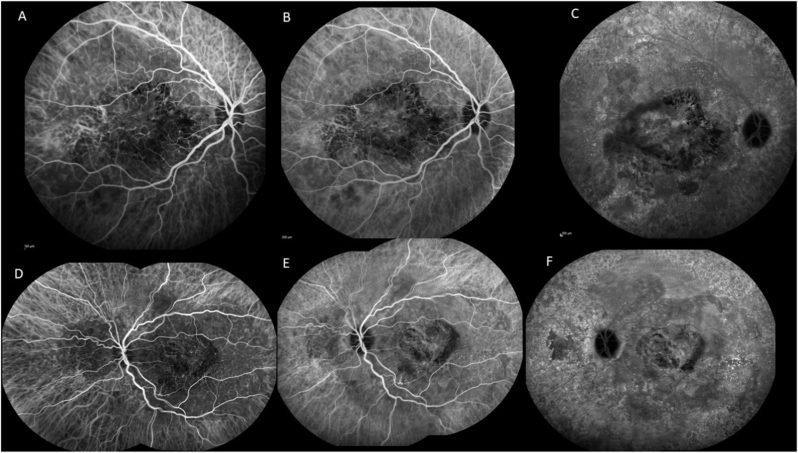
On indocyanine green angiography, the mass masked the underlying choroidal cyanescence in the early (A), mild (B) and late phases (C) of the right eye and the left eye (D, E, F).

**Fig. 7:**
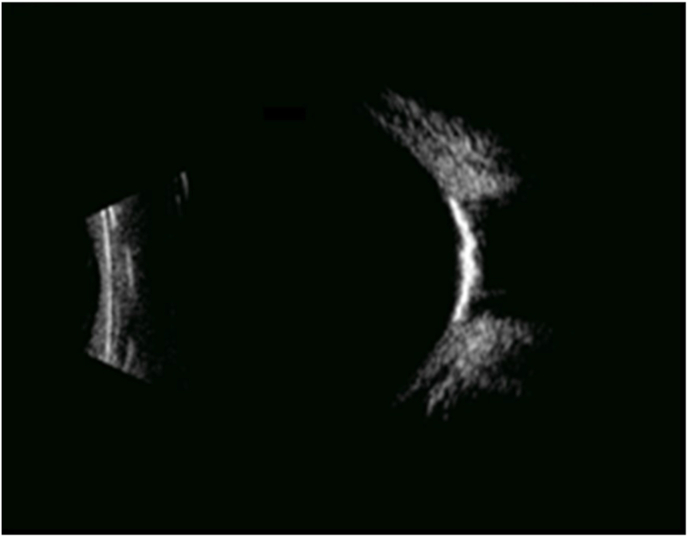
B-scan ultrasonography showed a sharply demarcated hyperechoic choroidal lesion with posterior shadowing.

Six years later, visual acuity had decreased to 20/125 for the right eye and 20/50 for the left eye. Fundus examination revealed yellowish choroidal infiltration with RPE elevation, pale atrophic areas, hyperpigmentation, and scarring at the posterior pole, associated with subretinal fibrosis in both eyes (Fig. 3A) and an intraretinal hemorrhage in the left eye (Fig. 3B). Fundus autofluorescence demonstrated hypoautofluorescent lesions due to the masking effect of RPE elevation and the surrounding hyperautofluorescence (Fig. 3C and D). Fluorescein angiography revealed heterogeneous early hyperfluorescence and staining of the choroidal infiltration in the right eye (Fig. 4, Fig. 5A), and leakage from choroidal neovascularization in the left eye (Fig. 4B and D). Optical coherence tomography (OCT) demonstrated a dome-shaped hyperreflective subretinal lesion, a thickened and disorganized choroid in both eyes, and an enlarged Haller vein bordering the infiltrate (Fig. 5B). In the left eye, subretinal fluid and hyperreflective subretinal exudation were observed at the site of choroidal neovascularization. Retinal nerve fiber layer (RNFL) thickness remained within the normal range. Indocyanine green angiography showed hypofluorescence of the lesion, persisting throughout the examination (see Fig. 6). B-scan ultrasonography showed a markedly hyperechogenic lesion with posterior shadowing (Fig. 7).

A review of the patient's medical history revealed follow-up since the age of four years for genetically confirmed HS with biallelic pathogenic variants of *SLC29A3*, presenting as pigmented scleroderma of the lumbar area and lower limbs, growth retardation, bilateral sensorineural hearing loss, camptodactyly, and persistent inflammatory syndrome. Several immunosuppressive therapies (corticosteroids, mycophenolate mofetil, anakinra, infliximab, methotrexate) had been attempted over the years, resulting in cutaneous remission.

On examination as an adult, the patient displayed open-lattice reticular livedo without induration, necrosis or scleroderma. Laboratory tests showed an inflammatory syndrome with a C-reactive protein (CRP) concentration of 30 mg/L. Magnetic resonance imaging (MRI) revealed a contrast-enhanced infiltration of epicardial fat in the right ventricle and a fatty degeneration of extraocular muscles. Positron emission tomography/computed tomography (PET/CT) revealed a pathological hypermetabolism of infiltrative lesions in the thoracic prevertebral space, pericardium, renal fossae and subcutaneous tissue of the buttocks and thighs, without long-bone involvement.

A biopsy of perirenal fat showed fibroadipose tissue infiltrated principally with CD68^+^ CD163^+^ CD4^+^ CD14^+^ CD1a^–^ Langerin^–^ histiocytes. Immunohistochemical staining for BRAF V600E was negative. The infiltrate also included small CD3^+^ T lymphocytes and scattered CD20^+^ B cells. No mutations affecting proteins of the mitogen-activated protein kinase (MAPK) pathway were detected on molecular analysis.

The overall clinical, radiological, and histopathological features suggested an ECD-like phenotype; however, in the context of genetically confirmed SLC29A3-related disease, the findings were ultimately considered part of the broader spectrum of SLC29A3-associated histiocytosis (sometimes referred to as histiocytosis-lymphadenopathy plus syndrome).

The patient received three-monthly injections of ranibizumab (0.5 mg/0.05 ml), leading to a resolution of intraretinal fluid and hyperreflective subretinal exudation. Systemic therapy with cobimetinib, a mitogen-activated protein kinase (MEK) inhibitor, was initiated. Pathological hypermetabolism on PET/CT decreased or resolved. Visual acuity and the retinochoroidal lesion remained stable over two years of follow-up. The thickness of the choroidal mass in the nasal quadrant decreased from 642 μm to 349 μm after one year of treatment (Fig. 5C).

## 3 Case 2

The patient's 33-year-old brother, also known to carry biallelic pathogenic variants of *SLC29A3* but previously asymptomatic, underwent systematic ophthalmological evaluation in light of his sister's condition. His medical history included a presacral pseudotumor at the age of 29 years, initially diagnosed as paucicellular fibrous tissue and not followed further.

**Fig. 8:**
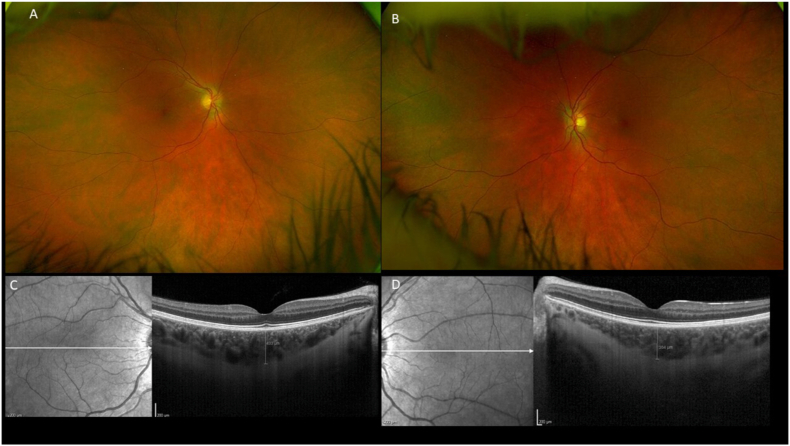
Fundus photographs of the right eye (A) and left eye (B) were unremarkable. Subfoveal choroidal thickening was present in both eyes (433 μm in OD and 382 μm in OS), with an enlargement of Haller's layer.

Ophthalmological examination revealed a visual acuity of 20/20 for both eyes. Fundus examination was normal. Choroidal infiltration with subfoveal thickening and an enlarged Haller layer were observed on OCT-B scan (Fig. 8)

Concomitant findings included a persistent inflammatory syndrome, bilateral perirenal and retroperitoneal infiltration complicated by hydronephrosis and end-stage renal failure, and pericarditis with tamponade. PET/CT revealed diffuse pathological hypermetabolism in the subcutaneous tissue, pericardium, and perirenal space. No long-bone involvement was detected.

A perirenal fat biopsy revealed fibrous connective tissue with rare CD68^+^ CD1a^–^ CD14^+^ S100^–^ histiocytes, and an absence of BRAF V600E mutation. However, strong phospho-ERK positivity was observed. No MAPK pathway mutations were detected on molecular analysis. A re-evaluation of the presacral biopsy performed in 2021 retrospectively confirmed non-Langerhans cell histiocytosis.

The systemic presentation was again suggestive of an ECD-like phenotype but was ultimately classified as SLC29A3-related histiocytosis. The patient was treated with cobimetinib. Subfoveal choroidal thickness decreased from 433 μm to 319μm in the right eye and from 382μm to 263μm in the left eye one year later.

## 4 Discussion

A literature review identified no previously published reports of familial SLC29A3-related histiocytosis with bilateral choroidal infiltration. However, one case of H syndrome associated with bilateral choroidal osteomas has been reported.^14^ The multimodal imaging features of choroidal osteoma may closely resemble those of choroidal infiltration, and histological examination remains the only method for definitively distinguishing between these entities. In our cases, choroidal osteoma was considered as a differential diagnosis but was deemed unlikely, as the decrease in choroidal thickness observed under MEK inhibition would not be expected in an osteomatous process, particularly in the context of histologically proven systemic histiocytosis.

Ophthalmological involvement is not a classical feature of H syndrome,^4^^,^^8^ but it is a recognized, albeit rare, manifestation of Erdheim-Chester disease, typically presenting as orbital pseudotumors or optic nerve compression.^9^ Choroidal infiltration was initially reported only sporadically in case reports of ECD^11^^,^^14^, ^15^, ^16^, ^17^, but was subsequently identified more frequently, in up to 16% of non-Langerhans cell histiocytoses, in systematic multimodal imaging investigations.^10^

Choroidal infiltration appears to be favored by the rich vascularization of this tissue and its potential role in extramedullary hematopoiesis. This may explain why myeloid-derived histiocytoses consistently involve the choroid, regardless of their genetic driver or histological subtype.^10^

The histological and radiological findings for both siblings — combined with their biallelic pathogenic variants of *SLC29A3* and a lack of BRAF V600E mutation — support the interpretation of SLC29A3-related histiocytosis with systemic and ocular involvement, mimicking certain features of Erdheim–Chester disease. It was not possible to identify the precise pathogenic SLC29A3 variants because genetic testing was performed in childhood and the original paper records are no longer accessible, in accordance with institutional archiving policies.

Our understanding of the pathophysiological mechanism underlying the transition from H syndrome to an Erdheim-Chester–like phenotype has recently improved. ECD is classically defined as a clonal histiocytosis driven by somatic mutations of MAPK pathway genes,^12^ whereas H syndrome is caused by recessive germline mutations of the *SLC29A3* gene, which encodes equilibrated nucleoside transporter 3 (ENT3).^1^^,^^2^^,^^6^ ENT3 normally facilitates the export of nucleosides from lysosomes into the cytoplasm; its dysfunction results in nucleoside accumulation and lysosomal impairment.

Shiloh et al.^6^ have provided compelling evidence that this nucleoside accumulation persistently activates lysosomal Toll-like receptors (TLR7 and TLR8), which in turn stimulate MAPK signaling, via the MEK–ERK axis in particular. This cascade promotes the transcription of inflammatory cytokines (IL-6, CCL2, CCL4, CXCL8), induces phospho-ERK^+^ histiocyte proliferation, and drives tissue infiltration. Importantly, histiocytic lesions in patients with H syndrome were positive for phospho-ERK despite the absence of somatic MAPK mutations, establishing a direct link between ENT3 deficiency and MAPK pathway activation.

This mechanistic parallel between germline ENT3 loss and somatic MAPK activation in ECD explains the clinical and histological convergence observed in our patients. The observed response to MEK inhibition further supports the idea that H syndrome lies within a broader *SLC29A3*-related histiocytosis spectrum,^18^ in which chronic innate immune stimulation via TLR–MAPK signaling reproduces the phenotype of clonal histiocytic neoplasms.

## 5 Conclusion

These observations support the concept of a unified pathophysiological spectrum of *SLC29A3*-related histiocytosis, in which choroidal infiltration may be an underrecognized ophthalmological feature.

## CRediT authorship contribution statement

**Xavier Boulu:** Writing – review & editing, Writing – original draft, Conceptualization. **Gilles Morin:** Writing – review & editing. **Christophe Attencourt:** Writing – review & editing. **Jean Schmidt:** Writing – review & editing. **Thi Ha Chau Tran:** Writing – review & editing, Writing – original draft.

## Patient consent

Written informed consent was obtained from both patients for the publication of anonymized clinical data and images.

## Funding

The study did not receive any specific funding from public, commercial, or not-for-profit agencies.

## Declaration of competing interest

The authors declare that they have no known competing financial interests or personal relationships that could have appeared to influence the work reported in this paper.

## Abbreviations

HS: H syndrome
ECD: Erdheim-Chester disease
OCT: Optical coherence tomography
RNFL: Retinal nerve fiber layer
PET/CT: Positron emission tomography/computed tomography
MRI: Magnetic resonance imaging
CRP: C-reactive protein
MAPK: Mitogen-activated protein kinase
MEK: Mitogen-activated protein kinase
ICG: Indocyanine green angiography
VEGF: Vascular endothelial growth factor
